# Receptor-Targeted Glial Brain Tumor Therapies

**DOI:** 10.3390/ijms19113326

**Published:** 2018-10-25

**Authors:** Puja Sharma, Waldemar Debinski

**Affiliations:** Brain Tumor Center of Excellence, Department of Cancer Biology, Wake Forest University School of Medicine, Comprehensive Cancer Center of Wake Forest Baptist Medical Center, 1 Medical Center Boulevard, Winston-Salem, NC 27157, USA; psharma@wakehealth.edu

**Keywords:** Glioblastoma, IL-13RA2, targeted therapies, tumor-associated receptor, peptide vaccines, dendritic cell-based vaccines, CAR T-cell therapy, eph/ephrin receptor system, EGFR/EGF receptor system, immune checkpoint inhibitors, viral/genetic therapies

## Abstract

Among primary brain tumors, malignant gliomas are notably difficult to manage. The higher-grade tumors represent an unmet need in medicine. There have been extensive efforts to implement receptor-targeted therapeutic approaches directed against gliomas. These approaches include immunotherapies, such as vaccines, adoptive immunotherapy, and passive immunotherapy. Targeted cytotoxic radio energy and pro-drug activation have been designed specifically for brain tumors. The field of targeting through receptors progressed significantly with the discovery of an interleukin 13 receptor alpha 2 (IL-13RA2) as a tumor-associated receptor over-expressed in most patients with glioblastoma (GBM) but not in normal brain. IL-13RA2 has been exploited in novel experimental therapies with very encouraging clinical responses. Other receptors are specifically over-expressed in many patients with GBM, such as EphA2 and EphA3 receptors, among others. These findings are important in view of the heterogeneity of GBM tumors and multiple tumor compartments responsible for tumor progression and resistance to therapies. The combined targeting of multiple receptors in different tumor compartments should be a preferred way to design novel receptor-targeted therapeutic approaches in gliomas.

## 1. Glial Tumors

In the nineteenth century, glial cells were perceived to hold the central nervous system (CNS) together. Hence, the word ‘glia’ arises from the Greek word, ‘glue’ [1]. Glial cells are supportive cells of the central nervous system. Gliomas arise when these supportive glial cells (or neuronal stem cells) [2,3,4,5] become malignant. The severity of malignancy is categorized in terms of grades. Several pathological features, such as abnormal appearance of the nucleus (nuclear atypia), necrosis, microvascular proliferation, and hemorrhages determine the extent of tumor aggressiveness and prognosis [6,7]. Lower grade (I–II) gliomas (LGG) include ependymomas, plilocytic astocytomas, angiocentric gliomas, papillary glioneural tumors, pituicytomas, choroid plexus carcinomas and rosette-forming glioneural tumors of the fourth ventricle. The latter exhibit more benign features and are associated with a better prognosis [8,9,10].

Unlike higher-grade gliomas, LGG do not usually show contrast enhancement after gadolinium injection on magnetic resonance imaging (MRI) scans; they also have ill-defined margins and appear heterogeneous on radiographic scans [6,8,10,11]. Their various morphologic patterns may not always coincide with specific clinical outcomes because their rate of growth varies. Some defined features of LGG include elongated cells, honeycomb patterns with absence of perinuclear staining, absence of mitotic figures, presence of glial fibrillary acidic protein (GFAP) and calcification, mutations in the isocitrate dehydrogenase gene (*IDH1*) [12], layers of tumor cells surrounding blood vessels forming pseudo-papillary structures and presence of perivascular pseudo-rosettes [6,8,13]. In over 70% of cases, *IDH1* mutations are caused by a single nucleotide substitution at the 132th amino acid from arginine (R) to histidine (H). LGG with *IDH1* mutations without co-deletion of 1p and 19q carry a relatively favorable prognosis. However, LGG without *IDH1* mutations are associated with the worst prognoses [14]. 

On the other hand, higher-grade (III-IV) gliomas exhibit more malignant features. Grade III gliomas include anaplastic gliomas [15], whereas Grade IV glioma is represented by glioblastoma (GBM) [16,17]. GBM is characterized by vascular proliferation, necrosis, pseudopalisading features [18], mitoses, and cellular and nuclear atypia [19].

GBM is also characterized by distinct molecular signatures and genetic mutations. Chromosome deletions of 1p and 19q, co-deletions of 1p and 19q [20,21] and mutations in telomerase reverse transcriptase (*TERT*) [22] are among those present in GBM. While single deletions of 1p and 19q represent an astrocytic lineage, co-deletions of 1p and 19q indicate an oligodendrocytic lineage and respond best to alkylating agent-based radiation therapy or chemotherapy like temozolomide [6]. *IDH1* mutations are also present in a small group of tumors called secondary GBM. *IDH1* mutations are associated with longer overall survival and progression-free survival among patients with GBM [23].

The Cancer Genome Atlas project first analyzed genomic changes in GBM. There are four major subtypes based on the assessment of mutational changes in 601 genes in GBM: (i) the proneural subtype, which occurs in younger patients with GBM, shows an oligodendrocytic lineage and is characterized by enhanced mutations in tumor protein 53 (*TP53*) and *IDH1* genes. (ii) The neural subtype, which occurs in older patients with GBM, has an astrocytic lineage without particularly high or low rates of mutation within specific genes. (iii) The classical subtype is characterized by lack of *TP53* mutations and enhanced expression of epidermal growth factor receptor (*EGFR*). (iv) The mesenchymal subtype is characterized by mutations in neurofibromin 1, phosphatase and tensin homolog (*PTEN*) and *TP53* genes, and shows an astroglial lineage [23,24,25]. Even though this division was proposed, it appears that nearly every GBM tumor exhibits the features of various subtypes, further indicating high levels of intra- and intertumoral heterogeneity [26]. 

Proposed in 2010, the Response Assessment in Neuro-Oncology criteria (RANO) is the standard method to assess the response to treatments in GBM. Assessment of treatments is largely based on contrast enhancing computerized axial tomography (CT) or MRI images. The RANO criteria are an improvement over the previously used Macdonald criteria, because they incorporate LGG or smaller tumor components of GBM. The user variability in CT and MRI scans, and evaluation of pseudo-progression after GBM treatment are also lesser concerns with the RANO criteria [27]. 

GBM accounts for 15% of all primary brain tumors, and represents 56% of all gliomas. About 12,670 new cases of GBM are expected to be diagnosed in 2018 in the U.S. alone [28]. Primary treatment for GBM includes surgical resection of the tumor, chemo therapy and radiation therapy often provided in combination. As a standard therapy, an alkylating agent like temozolomide is given during and after radiation therapy, especially to patients with promoter methylation of the DNA-damage repair protein O6 methylguanine-DNA methyltransferase (MGMT) [29].

Some major challenges associated with GBM treatment include poor penetration of drugs across the blood-brain barrier and the tumor; location of the tumor; infiltrative tumor cells migrating away from the primary tumor into normal brain parenchyma; resistance to chemotherapy and radiation therapy; faulty but extremely well-developed neovasculature; and molecular, genetic and morphological heterogeneity [30,31,32]. While a complete surgical resection of tumors is difficult due to tumor infiltration and proximity to critical motor/sensory nerves, molecular resection via selective targeting of glioma cells might be possible. 

Despite relentless research and clinical efforts, over the last eight decades, only one month per decade has been added to survival rates among patients with GBM [32,33]. In particular, the median survival of GBM patients treated with radiation therapy and surgery in 2000–2003 was 12 months, which increased to 14.2 months in 2005–2008 likely due to addition of temozolamide [34]. Today, the median survival of patients with GBM is 15–16 months for those who receive surgery, radiation therapy, chemotherapy, and tumor-treating fields (TTFields) [35]. In this review article (Box 1), we discuss targeted therapies against gliomas with a particular focus on receptors.

Box 1 **An overview of the review paper.**

- Glial Tumors Overview
  ○ Glial tumors are malignancies of supportive glial cells.
  ○ Low Grade Gliomas (LGG) overview: morphological, radiographic, molecular, and genetic characteristics
  ○ High Grade Gliomas (HGG) overview: morphological, radiographic, molecular, and genetic characteristics
  ○ Four major subtypes of GBM: proneural, neural, classical, and mesenchymal
  ○ Response Assessment in Neuro-Oncology (RANO) as a standard method used to assess treatment responses.
  ○ Major challenges in treatment of GBM: poor penetration of drugs, blood-brain barrier, location of the tumor, infiltrative nature of cells, heterogeneity, and resistance to therapy
- Targeted Therapies for Glioma
  ○ There is a need to specifically target glioma cells and spare normal cells.
  ○ Tumor Specific Antigens/ Tumor Associated Antigens (TSA/TAA) are specific to tumor cells and can serve as excellent targets for therapy.
  ○ Immunotherapy is the fourth pillar of cancer treatment in addition to surgery, chemotherapy and radiation therapy.
- Interleukin 13 Receptor Alpha 2 (IL-13RA2) in Glioma Treatment
  ○ Overexpressed in up to 75% of patients, IL-13RA2 was discovered as a glioma-specific marker over two decades ago in the Debinski Laboratory.
  ○ Over time, about 30 variations of targeted therapies have focused on IL-13RA2, including ICT 107 and IL-13-PE38QQR.
  ○ IL-13RA2 belongs to cancer/testes antigen group.
  ○ Over 200 cancer/testes antigens belonging to 70 different families have been identified so far.
  ○ Other cancer/testes antigens found in Glioma: ACTL8, XAGE3, BORIS, OIP5, DDX43, CTAGE1, KNL1, MAGE-A12, BAGE1
  ○ IL-13RA2 targeting:
  ○ IL-13 ligand can target IL-13RA2.
  ○ IL-13 based fusion/conjugated proteins, nanoparticles, liposomes, viruses, IL-13 conjugated radiotherapy agents, antibodies, peptide-pulsed dendritic cells, and CAR T-cells are used to target IL-13RA2.
  ○ IL-13PE38QQR, or Cintredekin Besudotox (CB) is one of the most potent recombinant cytotoxin in preclinical and clinical trials to treat glioma.
  ○ Optimizing the ligands for therapies:
  ○ Mutations in amino acid positions 13, 66, 69 and 105 can enhance the specificity of IL-13 to the IL-13RA2.
  ○ Addition of *Pseudomonas* exotoxin domain 2 (D2) and a nuclear localization signal (NLS) to IL-13 can directly transport IL-13 based cytotoxins to the nucleus of the glioma cells.
  ○ IL-13 based drug delivery:
  ○ IL-13 directed viruses: modified adeno and lentiviruses expressing IL-13 include LU-13 and MV Hcdelta18-IL-13.
  ○ IL-13RA2 targeted radiotherapy agents: copper-64 radiolabeled Pep-1L and alpha particle emitter, actinium-225 radiolabeled Pep-1L have been used to target IL-13RA2.
  ○ Peptide vaccines: multiple TSA/TAA including IL-13RA2, EGFRvIII, AIM-2, gp-100, HER-2, MAGE-1, EphA2, and survivin have been targeted using peptide vaccines.
  ○ Dendritic cell-based vaccines: multiple TSA/TAA including IL-13RA2, EphA2, Wilms tumor 1 have been targeted using multiple antigen pulsed dendritic cells.
  ○ CAR T-cell therapy: both first- and second-generation CAR T-cells have successfully targeted IL-13RA2 including a Phase I clinical trial for GBM at The City of Hope.
- Compromising a vastly interconnected tumor ecosystem with a single therapeutic approach is a great challenge. Hence, multiple targeting of various facets of the tumor ecosystem can enhance therapeutic potential and provide significant clinical benefit.
- Eph/ephrin receptor system in gliomas
  ○ EphA2, EphA3, and EphB3 are overexpressed in gliomas and have been extensively utilized for targeted therapy.
- EGFR/EGF receptor system in gliomas
  ○ EGFR/EGF is one of the most studied receptor systems in brain tumors.
  ○ *EGFR* is amplified in 40-60% patients and 30-50% *EGFR* amplifications contain *EGFRvIII* mutations.
  ○ EGFR targeted therapies include: siRNA, small molecule inhibitors, antibodies, and CAR T-cells.
- Other receptors/targeted therapies
  ○ Other receptors used to target glioma include: VEGFR, PDGFR, TGFR, FGFR, c-Met receptor, uPAR, GPCR, LDL-R, LRP1, and FOLR.
- Immune checkpoint inhibitors
  ○ GBM cells consistently work to maintain and generate an immunosuppressive environment.
  ○ While the use of immune checkpoint inhibitors shows great promise, its clinical benefits are yet to be established in glioma.
- Viral/genetic therapies
  ○ Over 20 oncolytic virus candidates including reovirus, oncolytic measles, oncolytic herpes simplex virus, and vesicular stomatitis virus have been tested in clinical trials to treat GBM.
  ○ While the use of viral therapy in glioma shows great promise, its consistent clinical benefits are yet to be established.
- Conclusions
  ○ Effective treatment of malignant gliomas is a great challenge in oncology.
  ○ Notable advancements in antigen-specific targeting, viral and genetic silencing, immune checkpoint inhibitors, peptide and dendritic cell vaccines, CAR T-cell therapy, chemotherapy and radiation therapy, in conjugation with effective drug delivery systems like CED offer promise to tackle this grave challenge in glioma therapy.
  ○ Targeting multiple facets of the tumor ecosystem, personalized antigen screening, and using state of the art *ex vivo* technologies can enhance the clinical benefits of current and future treatment options.

## 2. Targeted Therapies for Gliomas

**Figure 1:**
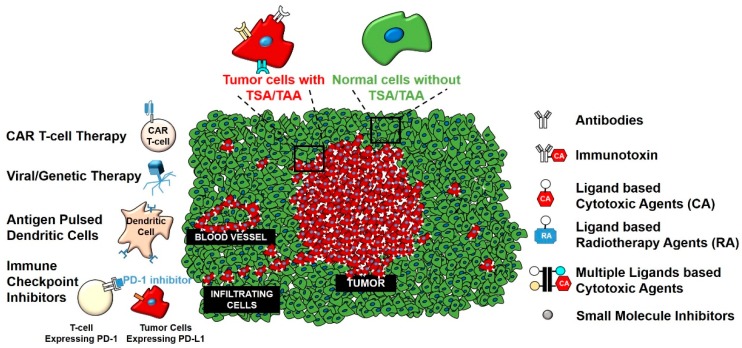
A schematic of various targeted therapies used to treat glioblastoma. Tumor cells express Tumor Specific Antigen/ Tumor Associated Antigen (TSA/TAA) that are lacking in healthy neighboring normal cells. These therapies specifically target the TSA/TAA to limit the cytotoxic/radio therapeutic effects to tumor cells and spare their normal counterparts.

The discovery of tumor-specific and tumor-associated antigens (TSA and TAA, respectively) have permitted development of a new array of therapeutic methods that selectively target gliomas and spare non-diseased cells of the CNS [36,37]. Many of these antigens are plasma membrane receptors [38,39]. Strategies to target TSA/TAA involve using antibodies to block ligand/receptor interactions, antibodies conjugated with modified versions of proteinaceous toxins or cytotoxic drugs or labels and nanoparticles or liposomes loaded with drugs that selectively target glioma TSA/TAA [40,41]. Immune checkpoint inhibitors that interact with a subset of T cell receptors have resulted in significant clinical effects in almost 10 cancers [42,43], to date, but not in brain tumors. Lastly, advances in immunotherapy treatments that involve peptide vaccines [44], antigen pulsed dendritic cells [45], tumor-infiltrating lymphocytes, and chimeric antigen receptor T-cells (CAR T-cells) have shown promise, but their clinical benefits in gliomas remain to be seen [46]. The U.S. Food and Drug Administration (FDA) recently approved CAR T-cell therapy, which targets cluster of differentiation (CD) 19^+^ cells in acute lymphoblastic leukemia (ALL) [47]. This is seen as one of the most powerful treatment options in oncology and potentially also for gliomas (Figure 1). This approach is being tested extensively for the treatment of GBM, as discussed below.

Immunotherapy has been recognized as the fourth pillar of cancer treatment after surgery, chemotherapy and radiation therapy [48]. The FDA’s approval of ipilimumab (a monoclonal antibody that targets cytotoxic T-lymphocyte-associated protein 4 (CTL-4) in melanoma) and sipuleucel-T (a personalized immunotherapy treatment for prostate cancers) encourages further immunotherapy research in glioma. While the benefit of immunotherapy has been well established in melanoma and prostate cancers [14,49], it is still being evaluated for the treatment of GBM and other astrocytic tumors. For example, a peptide-based vaccine plus poly-ICLC for low-grade gliomas using four targets (IL-13RA2, EphA2 receptor, Wilms tumor 1 and survivin) was well tolerated in patients with grade II glioma [50]. Patients exhibited significant increases in interferon γ (IFN γ) following vaccination. The median survival of patients was 17 and 12 months, respectively, for two cohorts, and one patient had progression-free survival for 67 months.

Traditionally considered an immune privileged system, recent research has shown that immune cells can cross the blood-brain barrier (BBB) and reach the brain parenchyma [51]. In addition, brain cells can perform the function of immune cells like microglial cells [52,53]. This line of research has opened novel avenues for new immunotherapeutic treatment opportunities for GBM [54,55,56,57,58,59]. This approach is gaining wider acceptance as reflected in the recent National Institute of Health (NIH) Cancer Moonshot Blue Ribbon Panel. The Panel had 10 transformative research recommendations, including ‘Create a translational science network devoted exclusively to immunotherapy’ [60]. One of the major challenges of these therapies is to selectively target antigens specific to gliomas. Henceforth, we will elaborate on TSA/TAA targeted at gliomas, and specifically membrane receptors that have been exploited to target therapies in laboratory, preclinical, and clinical settings.

## 3. Interleukin 13 Receptor Alpha 2 (IL-13RA2) in Glioma Treatment

**Figure 2:**
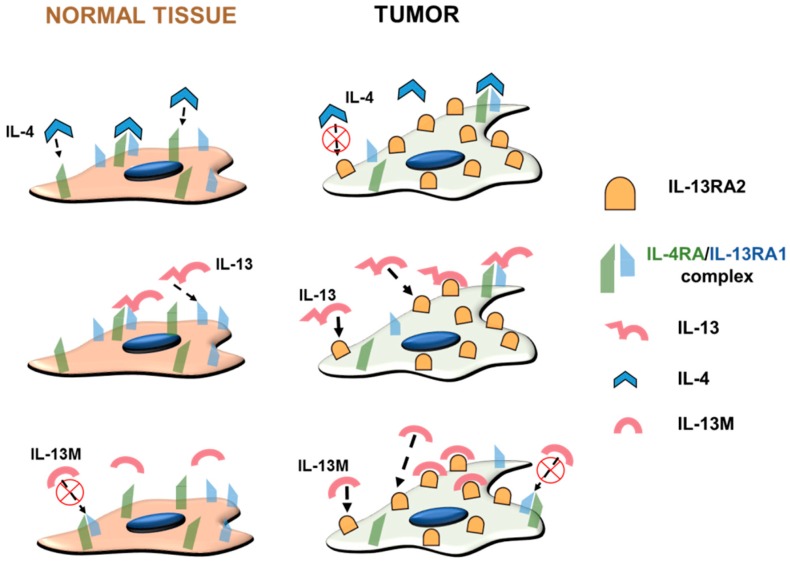
A schematic of the IL-13 receptor system. Normal and tumor cells express IL-4RA/IL-13RA1. IL-4RA/IL-13RA1 have IL-4RA (represented in green) and IL-13RA1 (represented in blue) subunits. IL-4 first binds to IL-4RA, then to the IL-13RA1 to form an IL-4RA/IL-13RA1 complex. IL-13 first binds to IL-13RA1, then to IL-4A to form an IL-4RA/IL-13RA1 complex in normal tissue. On the other hand, IL-13RA2 is expressed by tumor cells. IL-4 binds to IL-4RA/IL-13RA1, but not to IL-13RA2 (represented by a red encircled cross). IL-13 binds to both IL-4RA/IL-13RA1 and IL-13RA2. IL-13 binds more readily to IL-13RA2 compared to IL-4RA/IL-13RA1. By introducing mutation in the IL-13 ligand to produce IL-13M, the ligand binds mostly only to the IL-13RA2, and not to IL-4RA/IL-13RA1 complex (represented by a red encircled cross).

IL-13RA2 is one of the most thoroughly studied TAA in glioblastoma research. It was revealed as a glioma-specific marker over two decades ago in the Debinski laboratory, when a cytotoxic ligand-toxin fusion protein based on interleukin 13 (IL-13) demonstrated extreme potency against glioma cells (Figure 2) [61,62]. It is overexpressed in up to 75% of glioma patients [63]. Over the years, overexpression of IL-13RA2 in GBM has become a subject of intense interest by multiple laboratories, confirming IL-13RA2 as a powerful target for anti-glioma therapies. Over 30 variations of treatments focused on IL-13RA2 have been used to target and kill glioma cells in vitro, preclinical, and clinical settings [64]. CAR T-cells targeting IL-13RA2 are now being tested in a Phase I clinical trial [56]. 

One promising example is ICT-107, which is an autologous dendritic cell-based vaccine pulsed with six different antigens: IL-13RA2, melanoma associated antigen 1 (MAGE-1), tyrosine-related protein 2 (TRP-2), glycoprotein 100 (gp100), epidermal growth factor receptor (HER-2), and absent in melanoma 2 (AIM-2). However, a Phase III clinical trial of ICT-107 (NCT02546102) was suspended by ImmunoCellular due to the lack of funding to complete the trial. Nonetheless, progression-free survival was significantly improved in the Phase II trial. 

The completion of a Phase III trial for IL-13-PE38QQR, the first generation of IL-13-based cytotoxins, showed good tolerance of the drug candidate with promising effects [65]. Unfortunately, the trial sought to extend the survival of patients with recurrent GBM by 50% compared to historical controls. Virtually no drug in oncology has shown such activity. Hence, not surprisingly, the trial did not achieve the company’s chosen clinical endpoint. Nevertheless, the survival rate achieved in patients with recurrent GBM in this trial has not been improved or matched since.

IL-13RA2 is coded by the X chromosome, and is composed of 380 amino acids with a 17 amino acid intracellular domain and a 26 amino acid signaling sequence [64]. Gene expression of *IL-13RA2* in normal tissues is low to undetectable, but high in testis. Protein expression of IL-13RA2 is high in testis and placenta, but very low or undetectable in other organs. Male reproductive cells show characteristics that are advantageous to tumor cells, such as proliferation, migration, and evading immune responses. Hence, a number of antigens are commonly expressed in cancer cells and testes. Like IL-13RA2, an array of antigens described as the cancer/testis antigen group are commonly found in cancer and testis [66]. They are broadly classified into two groups, one coded by the X chromosome and the other coded by autosomes. MAGE-1 was the first of this group to be discovered, over 25 years ago [67]. Other cancer/testis antigens coded by the X chromosome include synovial sarcoma X (SSX), cancer/testis antigen 45 (CT45), and G-antigen (GAGE), whereas those coded by autosomes include B melanoma antigen (BAGE), sperm protein (SP17) and helicase antigen (HAGE). To date, over 200 cancer/testis antigens have been identified among 70 different families [66,68].

Some cancer/testis antigens overexpressed in glioma include actin like protein 8 (ACTL8), X-antigen family member 3 (XAGE3), brother of the regulator of imprinted sites (BORIS), and opa-interacting protein 5 (OIP5) [69]. Glioma cell lines derived from patients with glioma also exhibit overexpression of cancer/testis antigens, especially IL-13RA2, dead-box helicase 43 (DDX43), cutaneous T-cell lymphoma associated antigen 1 (CTAGE1), kinetochore scaffold 1 (KNL1), MAGE-A12 and BAGE l [70]. Higher expression of these antigens was associated with worse prognosis in patients. Some of the hypotheses that possibly explain the advantage of amplified cancer/testis genes in tumor cells are discussed below.

One hypothesis is that glioma cells evade immunosuppression via the overexpression of IL-13RA2. Interleukin 13 (IL-13) is primarily produced by T helper 2 (T_H_2) cells, and also by CD8^+^ T cells after activation, mast cells, basophils, and eosinophils [71]. It is believed to inhibit pro-inflammatory cytokines and play a role in anti-inflammatory responses. Overexpression of IL-13 has also been associated with immunosuppression [72] and low serum levels of IL-13 have been associated with poor overall survival in colorectal cancer [73]. IL-13RA2 is highly overexpressed in GBM, with up to 30,000 binding sites per cultured GBM cells [61]. The GBM cell line G48a expresses about 4,000,000 binding sites for IL-13RA2 per cell [74,75,76,77,78,79,80]. IL-13 binds to IL-13 receptor A1 (IL-13RA1), forms a more stable heterodimer with IL-4 receptor A (IL-4RA), and then activates downstream signaling pathways [62,74,81]. IL-13 binds more readily to IL-13RA2 than to IL-13RA1 to form a heterodimer with IL-4RA, making IL-13RA2 an IL-13 sequestering receptor in the GBM microenvironment. Hence, by sequestering the IL-13 available in the tumor microenvironment, GBM cells could be evading immunosuppression.

Another hypothesis is that by sequestering IL-13, GBM cells can escape apoptosis that ordinarily would have been activated by the signal transducer and activator of transcription 6 (STAT6) pathway as an aftermath of IL-13 binding to IL-13RA1/IL-4RA, which is also expressed on tumor cells to some extent. Hence, by taking IL-13 away from the system, GBM tumor cells could avoid apoptosis [82]. IL-13RA2 expression is also correlated with higher glioma grades and cells exhibiting a mesenchymal phenotype in glioma [83]. Eliminating IL-13RA2 positive cells can make tumors less tumorigenic and invasive [84]. Hence, the advantages of targeting IL-13RA2 seem to be threefold: (a) effective and selective targeting of glioma cells sparing normal brain tissue, as normal brain cells do not express IL-13RA2; (b) making IL-13 available for activating the heterodimeric receptor shared with IL-4 by avoiding sequestration; and (c) eliminating the more invasive mesenchymal components within the tumor population. 

### 3.1. IL-13RA2 Targeting

IL-13RA2 targeting agents are represented by IL-13-based fusion/conjugated proteins (e.g., to bacterial toxins), nanoparticles and liposomes, viruses, IL-13 conjugated radiotherapy agents, antibodies (e.g., conjugated to cytotoxic drugs, and unconjugated), peptide pulsed dendritic cells or receptor peptides, and CAR T-cells against IL-13RA2. These can be broadly categorized as types of active, passive, or adoptive immunotherapy. Active immunotherapy entails the arming of immune system to initiate long-term memory by allowing the immune system to fight tumor cells long after the administration of the therapy [85,86]. Passive immunotherapy on the other hand, is the administration of immunotherapeutic agents that are based on the elements of the immune system, temporary, but still could instill a long term memory under certain conditions [87]. Adoptive immunotherapy or adoptive cell transfer (ACT) involves the isolation of T-cells from patients. These T-cells are genetically modified to express TSA or TAA protein-binding moieties. Chemotherapy can then be administered to weaken the existing tumor environment. Finally, the genetically modified T-cells are introduced into a patient so that the engineered T-cells can selectively home and destroy the tumor cells of interest by recognizing TSA/TAA [88].

Recombinant cytotoxins are targeted to GBM as fusions/conjugates to an antibody or a ligand specific to a receptor. Upon ligand/receptor interaction, the toxin/drug is internalized only by tumor cells or elements of the tumor microenvironment expressing the given receptor. The first use of bacterial toxin to treat cancer was reported by Dr. William B. Coley in the 1890s to treat sarcoma [89]. However, due to inconsistent results, it was overtaken by more reliable therapies like chemo- and radiation therapies. One of the most potent recombinant cytotoxins in preclinical and clinical trials to treat glioma has been IL-13PE38QQR. This cytotoxin also led to the discovery of the IL-13RA2 overexpression in gliomas. This recombinant cytotoxin is based on wild-type IL-13 ligand, which binds both IL-13RA1/IL-4A and IL-13RA2 receptors, fused to a form of modified *Pseudomonas* exotoxin A (PE), PE38QQR [90]. The anti-tumor activity of IL-13-PE38QQR was further confirmed with intratumoral injections in animal models of GBM [91].

In commercial clinical development, IL-13-PE38QQR was called cintredekin besudotox (CB) [92]. CB was delivered by convection-enhanced delivery (CED) and was well tolerated by patients in Phase I/II clinical trials (maximum tolerated concentration 0.5 µg/mL). CB was infused at 0.75 mL/h divided by the number of catheters (up to four) for 96 h. Patients were followed for 30 days after treatment before the next set of patients was enrolled in the study. Some side effects included seizures, numbness, headaches, confusion, fatigue, repeated and uncontrolled eye movements, and gait changes. One of the severe side effects included hemiparesis [93]. 

In another study of 22 patients with newly diagnosed high-grade glioma, CB was also well tolerated. Only one patient exhibited a dose-limiting grade 4 seizure, and another experienced dose-limiting grade 3 confusion and aphasia, but no patients had grade 3 or 4 hematologic toxicities [94]. Overall, the best results were obtained when catheter positioning was monitored during CB infusion by MRI [93]. When the drug was delivered via more than two catheters in a MRI-guided setting, outcomes were improved [65,93,95]. The median survival of patients with more than two optimally placed catheters was 55.6 weeks, whereas that of patients with less than two optimally placed catheters was 37.4 weeks. Furthermore, suboptimally placed catheters were associated with leakage in the cerebrospinal fluid compartment [93].

A Phase III clinical trial (called the PRECISE study) was performed to compare overall survival and assess toxicity and safety of CB versus Gliadel wafers after the first recurrence of GBM [96]. Using 2 to 4 MRI-guided catheters, drug was delivered to targeted brain areas. The PRECISE study was performed in >50 centers across North America and Europe and involved 296 patients. Patients were randomized to receive CB or Gliadel wafers in a 2:1 ratio. Gliadel wafers were placed immediately after tumor resection. Among patients randomized to CB, 2 to 4 catheters were placed in areas most vulnerable to infiltrating tumors 2–7 days after tumor resection. After 24 h, CB was infused for 96 h at 0.750 mL/h at 0.5 µg/mL; CT scans were used to verify catheter placement. Participants were assessed for radiographic and clinical responses every 8 weeks after treatment. Patients treated at centers more experienced with catheter positioning and neurosurgery had better outcomes than those at centers where less than 2 patients were enrolled [97]. Similarly, most side effects were psychiatric or neurologic in nature and included headache, speech and gait disorder, nausea, sensory disturbance, facial paresis, and memory impairment. These could have been an aftermath of IL-13-PE38QQR binding to IL-13RA1 receptors in normal brain tissues. Patients were not screened for IL-13RA2 in their tumors as an eligibility criterion, and drug distribution was not monitored during prolonged infusion. Gene array studies involving 959 GBM samples have shown that *IL-13RA2* is over-expressed in 58% [83] and the protein is present up to 75% of GBM. Therefore, screening of patients for IL-13RA2 positivity could have helped to obtain better responses to CB.

Future considerations to make the targeted cytotoxin therapy more effective could be divided into clinical (methods of drug delivery improvement) and laboratory (ligand/drug improvement) settings. Clinical considerations could include real-time monitoring via MRI of the drug infusion using CED, proper positioning of the catheter through pre-infusion planning, potentially restricting the treatment to more experienced centers, and screening patients for targets before treatment. Overall, optimizing the binding of IL-13 to IL-13RA2 while minimizing its binding to the normal tissue/tumor IL-13RA1/IL-4RA complex could improve outcomes of IL-13RA2 targeting in GBM patients. 

### 3.2. Optimizing the Ligand for Therapies

Of the four alpha helices of the IL-13 ligand, helix D is primarily responsible for its interaction with IL-13RA2 [98]. Mutations such as replacing the 13th amino acid from glutamic acid (E) to lysine (K) to produce IL-13.E13K—or changing from glutamic acid (E) to tyrosine (Y) to produce IL-13.E13Y —could make the ligand more specific to IL-13RA2 by considerably decreasing its association with IL-4A and formation of the IL-13RA1/IL-4 receptor [61,99,100]. Additional mutations in arginine to aspartic acid, serine to aspartic acid, and lysine to arginine at positions 66, 69 and 105 (in α-helices C and D, respectively) enhance specificity of binding of the IL-13 ligand to IL-13RA2 [100,101]. 

The IL-13.E13K ligand has been incorporated in a single-chain protein with domain 2 (D2) of PE, and a nuclear localization signal (NLS) used to precisely deliver to nuclei the portion of the engineered protein produced by a proteolytic cleavage in an endocytic compartment [78,79,102]. A doxorubicin variant was also conjugated to the C-terminal end of the engineered protein and was toxic to glioma cells. Studies from the Debinski lab have shown that the protein was encapsulated by glioma cells in which IL-13RA2 was overexpressed, and the protein was transferred to the nucleus to deliver the cytotoxic load as planned [79,102]. 

Several antibodies for IL-13RA2 have been generated with high affinity to glioma cells in vitro [77,103]. Furthermore, even without conjugation to a bacterial toxin, the antibody enhanced survival of mice injected with glioma cells [103]. Thus, there is a possibility that conjugating antibody fragments to toxins may further enhance the cytotoxic effects of therapy on glioma cells. 

### 3.3. IL-13-Based Drug Delivery

Drug delivery systems armed with modified IL-13 to specifically target IL-13RA2 include (but are not limited to) liposomes, nanoparticles, and nanosheets. Liposomes range from 20 nm–3 µm in diameter and are composed of phospholipid lipid bilayers that allow simultaneous loading of hydrophobic and hydrophilic drugs. Liposomes offer many advantages for drug delivery. While modifications of the liposomal surface could allow decoration of various ligands to target different antigens in glioma, systemic delivery of liposomes is also possible. Liposomes directed by IL-13 significantly reduced tumors in glioma xenograft-bearing mice versus liposomes that were not guided by IL-13 [104,105]. Similarly, the liposomes directed by IL-13 and loaded with doxorubicin reduced tumor volume 5-fold, and mice survived more than 200 days; those that received non-targeted liposomal delivery of doxorubicin survived less than 35 days. Huang et al., also designed IL-13 peptide enabled mesoporous silica nanoparticles to deliver doxorubicin to glioma cells. In U-251 GBM cells that overexpress IL-13RA2, uptake of targeted nanoparticles was significantly higher compared to control nanoparticles [106,107]. 

#### 3.3.1. IL-13-Directed Viruses

Oncolytic virus-based treatment of glioma uses viruses engineered to induce tumor cell lysis, amplify therapeutic genes within the tumor cells, divide selectively within tumor cells, and/or also initiate immune response to themselves and dying tumor cells. Some oncolytic viruses used to treat GBM include herpes simplex virus, adeno- and lentivirus, measles virus, and polio virus. By modifying viruses to express ligands that more selectively target glioma cells, their side effects and toxicity are expected to decrease. For example, a Phase I clinical trial of 37 patients tested dose escalation and biologic endpoints of DNX-2401, an adenovirus that targets the α_v_β_3_ and α_v_β_5_ integrins in glioma cells via the arginylglycylaspartic acid peptide [108]. Twenty percent of patients survived >3 years, 2 patients had stable disease and 3 patients showed a complete response with >95% tumor reduction when 25 patients were treated with DHX-2401. Since DNX-2401 was well tolerated by patients, no dose-limiting toxicity was established. The maximum dose used (3 × 10^10^ virus particles (vp) in 1 mL) was formulated by manufacturing limitations, not by toxicity. 

Modified adeno- and lentiviruses that express the IL-13 (LU-13) and (MV Hcdelta18-IL-13) target glioma cells more than the control viruses [109,110]. Similarly, R5111, a herpes simplex virus that expresses IL-13, selectively targets glioma cells in vitro [111]. An attenuated measles virus targeting the HER2 receptor significantly prolonged survival of severe combined immunodeficiency (SCID) mice that contained ~35 mm^3^ tumors from HER2 positive SK-OV-3 xenografts. While tumor volumes for mice in the control group increased significantly, mice that received designed ankyrin repeat proteins (DARPins) measles virus showed complete remission [112]. Furthermore, adenovirus Ad.mhIL4.TRE.mhIL13PE co-expressed mutated ligand IL-4 (IL-4.Y124D) along with IL-13 conjugated to PE (mhIL13PE) [113] have also been designed. This strategy was introduced to abrogate the binding of IL-13 to the IL-13RA1/IL-4A receptor in normal cells, thus minimizing toxicity of the viral construct to normal brain and maximizing therapeutic efficacy. This approach helped to maintain strong anti-tumor activity of the targeted cytotoxin, with significantly decreased side effects compared to IL-13PE38QQR (CB) given alone [113].

#### 3.3.2. IL-13RA2-Targeted Radiotherapy Agents

Radioactive agents have been conjugated with peptides targeting the IL-13RA2 [78]. Pep-1L is a peptide ligand that binds to IL-13RA2 with high specificity. Using a phage display library and bioplanning schemes in glioma cells that overexpressed or did not express IL-13RA2, three peptide ligands were identified. Among them, Pep-1L showed the highest affinity to IL-13RA2, so it was used to home IL-13RA2 [78]. Copper-64 radiolabeled Pep-1L selectively bound to IL-13RA2-expressing cells in vitro [63]. Furthermore, labeled Pep-1L targeted GBM both in vitro and in an orthotropic model of GBM, monitored in real time using positron emission tomography (PET). This study also tested conjugation of Pep-1L with an alpha particle emitter, actinium-225. Using CED, [^225^Act] Pep-1L increased survival of mice compared to saline control. Treated tumor cells also showed lower amounts of Ki67 staining (proliferation marker) and higher internalization of propidium iodide in glioma cells, all of which indicate induction of apoptosis.

### 3.4. Peptide Vaccines

Peptide vaccines stimulate the immune response to selectively target antigens expressed in tumors. Neoantigens arise in tumors from somatic mutations and are absent or nearly absent in normal cells. Peptide vaccines could be composed of single or multiple peptides targeting single or multiple neoantigens or TAAs. TAAs specific to glioma that have been used to devise peptide vaccines include EGFRvIII, IL-13RA2, AIM-2, TRP-2, gp-100, HER-2 and MAGE-1 [114]. Although heterogeneous and sporadic, EGFRvIII has been proposed as a potential tumor-initiating cell and cancer stem cell marker [115]. The effects of an EGFRvIII-specific vaccine, Rindopepimut/CDX-110 (a keyhole limpet menocyanin conjugated with a 14 amino acid peptide that targets the deletional junction in EGFRvIII) were examined in 18 patients with GBM who had *EGFRvIII* mutations [116]. This vaccine was tested in conjunction with surgery and radiation therapy; treated patients survived 26 months on average. However, when tumors recurred, 82% of tumors did not expresses the *EGFRvIII* mutation likely due to immunoediting. A Phase III clinical trial was performed following earlier phases with negative final result, and did not show overall significant benefit [117]. 

Immunoediting occurs when the immune system continuously monitors tumor and eliminates cells that exhibit neoplastic mutations. Over time, single-peptide vaccines cause overgrowth of cells that do not express the targeted antigen. Hence, targeting multiple antigens at once should allow more robust treatment against glioma. A peptide vaccine targeting EphA2, IL-13RA2, and survivin was administered with tetanus toxoid and poly I:C in 26 children with low-grade gliomas (NCT01130077) and was well tolerated in children [118]. This peptide vaccine showed encouraging clinical outcomes, and no dose-limiting non-CNS toxicity was established. Nineteen patients showed stable disease, and temporary pseudo-progression was seen in 5 patients, along with edema (indicative of immune cell infiltration within the tumor) [119].

### 3.5. Dendritic Cell-Based Vaccines

Because dendritic cells can activate T cells, they have been used to make vaccines against tumor cells. Dendritic cells are isolated from blood of patients, pulsed with TAAs, and introduced back into the patients to try to activate CD4^+^ and CD8^+^ T cells and thus selectively target tumor cells expressing the antigens of interest. Dendritic cells pulsed with IL-13RA2 peptides increased immune response among glioma patients [120]. Patients with LGGs who were treated with multiple antigen-presenting dendritic cells, including EphA2, IL-13RA2, Wilms tumor 1 and survivin, also had increased immune responses [50]. Another Phase II multicenter study (NCT01280552) tested the use of dendritic cells (ICT-107) pulsed with 6 different antigens, including IL-13RA2 in patients with glioma. Progression-free response was increased with treatment, and among the 22 patients who received the vaccine, 58% had a positive response [114].

### 3.6. CAR T-Cell Therapy

The idea of using immune cells to fight tumor cells was introduced in the 1980s by Dr. Steven Rosenberg’s group at the NCI, who pioneered the extraction of tumor- infiltrating lymphocytes to treat melanoma [121]. Later, they designed T cell receptors to target melanoma-associated antigen recognized by T cells (MART-1) in patients with malignant melanoma [122]. This treatment caused regression of tumor growth in patients after ACT, but there were difficulties in extraction and few tumor-infiltrating lymphocytes were isolated from tumor sites. In addition, T-cell recognition depended on major histocompatibility complex (MHC), which limited its use. However, CAR T-cells recognized TSAs independent of MHC [123,124]. CAR T-cell therapy involves isolation of white blood cells of the patient, activation of T-cells using artificial dendritic cells, transduction with the CAR gene using a viral vector, expansion, and finally delivery back into patients. Before receiving CAR T-cells, patients can undergo chemotherapy to suppress normal white blood cells that could interfere with efficacy of the CAR T-cells [125].

The first FDA-approved CAR T-cell therapy, Kymriah, was developed to target CD19^+^ cells for patients with ALL [126]. Kymriah is a second generation CAR T-cell therapy that contains anti-CD19 single chain variable fragment (scFv) fragment, a transmembrane domain, and co-stimulatory domains CD3ζ and CD137. It was reported that 63% of patients showed complete remission, with no traces of blood markers that indicate a relapse 3 months after therapy. Very recently, the FDA also approved a second CAR T therapy (Yescarta) for adult patients with relapsed B-cell lymphoma [127]. Similar to Kymriah, Yescarta also targets CD-19. Currently, many CAR T-cell based therapies are being evaluated for various types of cancers. This approach offers further promise for effective treatment of cancer using the body’s own immune cells.

Both first and second-generation CAR T-cell therapies have been successfully used to target IL-13RA2. These CAR T-cells expressed the mutated IL-13 ligand (IL-13. E13Y) to enhance binding to IL-13RA2 [56,128,129,130,131,132]. IL-13. E13Y CD8^+^ T cells, which are an example of the first-generation of CAR T-cells, significantly affected cell viability in U-251 GBM cells in vitro and in vivo [129]. The anti-tumor response was also extended to glioma stem-like cells. Also, elevated secretion of immunostimulatory cytokines, including tumor necrosis factor α (TNFα) and INF γ was observed when the IL-13 zetakine^+^ CD8^+^ T-cells were co-cultured with U251 cells [133]. The City of Hope group conducted a Phase I clinical trial for GBM, using CAR T-cells against IL-13RA2 [46,56]. A patient with recurrent multifocal GBM who had failed all available therapies received CAR T-cells against IL-13RA2; complete regression was observed of all spinal and intracranial lesions, with no exhibit toxic effects greater than grade 3. This clinical response was coupled with an increase in immune cells and cytokines and lasted for at least 7.5 months after initial therapy [56]. In another study, 3 patients with recurrent glioma received first-generation CAR T-cells against IL-13RA2, which also included herpes simplex virus type 1-thymidine kinase and the PET reporter gene to non-invasively detect CAR T-cells on PET imaging [134,135]. IL-13. E13Y-zetakine/HyTk^+^ CD8^+^ T cells were present at the sites of injection and recurrence [135,136]. In another Phase I clinical trial (NCT01082926), IL-13-zetakine/HyTK CAR first-generation CAR CD8^+^ T-cells were expressed in CD8^+^ T cells from a healthy donor. These cells were co-administered with interleukin 2 (IL-2) and showed anti-tumor effects in patients with GBM expressing IL-13RA2. These promising results indicate that CAR T-mediated therapy holds potential as a treatment for glioma [64,137].

Nonetheless, one issue with CAR T cell therapy is that the survival and persistence of targeted T-cells in the glioma environment is limited. Using second-generation CAR T-cells with IL-13.E13K.R109K and a co-stimulatory signal CD28 and CD3ζ, Kong et al. reported increased survival using in vivo xenograft models [138]. Along with longer survival, CAR T-cells displayed higher affinity to IL-13RA2 compared to IL-13RA1 in normal tissue, along with significant increased immune responses, including secretion of immunostimulatory cytokines such as IL-2 and INF γ. Ways to improve the efficacy of CAR T-cell therapy could include enhancing the affinity of IL-13 ligand to IL-13RA2, and introduction of the IL-15 gene [139,140,141], IL-12, and nuclear factor of activated T-cells (NFAT), to boost survival and proliferation of T-cells within tumors. 

While CAR T-cell therapy has shown encouraging anti-tumor results, challenges remain to improve its clinical efficacy. For example, the Phase II ROCKET trial was terminated prematurely after 5 of 38 patients receiving the treatment died after developing neurotoxicity and brain swelling following treatment [142]. Additionally, CAR T-cell activation usually accompanies elevated levels immunostimulatory cytokines, leading to the cytokine release syndrome [143]. An inhibitor of interleukin 6 (IL-6), Actemra (tocilizumab), is used to address this issue [144]. However, a better alternative would be a potential genetic switch within the CAR T-cell system that could turn off when excess cytokines are being released. Companies like Cellectis and Bellicum Pharmaceuticals are experimenting with UCART 19 [145] and GoCAR T-cells that are activated in the presence of rapamycin and rimiducid, respectively [146]. Similarly, efforts are being made to house caspase-9 based switches in CAR T-cells to target CD20^+^ cells in lymphoma [147].

Another significant challenge for successful CAR T-cell therapy, or immunotherapy in general, is the loss of a targeted antigen with treatment. Studies of CAR T-cell therapy have suggested that decreased expression of IL-13RA2 could be a factor in tumor recurrence [56]. Loss of EGFRvIII has also been described after CAR T-cell therapy [148]. This has been called ‘antigen-negative immune escape’ [149] and might be caused by unintentional selection of antigen negative cells that proliferate faster than the antigen-positive cells within the tumor. It could also be caused by loss of antigen within tumor cells that previously expressed the antigen. Identifying the mechanism for immunoediting could help design improved CAR T-cells to minimize this unintended effect and eventually reduce therapeutic resistance.

Furthermore, simultaneously targeting of different antigens could enhance CAR T-cell therapy by making loss of antigens less likely [150]. T and CAR T-cells that target HER2 and IL-13RA2 have been tested in a murine model of GBM, and was associated with less antigen escape and significantly improved survival [150]. But depriving tumors of IL-13RA2-expressing cells might have its own beneficial effects, because these cells took the longest to show tumor recurrence [151]. This result suggests that tumor cells lacking IL-13RA2 might be less tumorigenic, which was actually seen in cells selected for IL-13RA2 down-regulation [152].

Resistance to immunotherapy can be inherited, as when a cancer does not respond to therapy in the first place; or it can be acquired, where a tumor initially responds to the therapy, but over time becomes resistant [153]. This resistance can result from various intrinsic factors such as phosphatase and tensin homolog deleted on chromosome 10 (*PTEN*) loss [154], the mitogen-activated protein kinase pathway (MAPK) [155], and extrinsic factors such as recruitment of immunosuppressive cells like regulatory T cells, myeloid-derived stem cells, and loss of T cells over time specific to certain tumor antigens [153,156]. Given the failure of many monotherapies in Phase III clinical trials, tumors should always be viewed as an ecosystem with multiple interconnected heterogeneous layers that feed off each other by sustaining a supportive biochemical and biophysical microenvironment along with immune system and tumor cells [26,157,158,159].

In the tumor ecosystem, identification of ‘keystone species’ that essentially hold the ecosystem together is extremely difficult, and has not been identified in GBM. It is therefore not surprising to observe tumor recurrence, just as an ecosystem would recover after the loss of one species. Hence, therapeutic approaches to GBM should also acknowledge the complexity of the tumor ecosystem to gain significant clinical benefit. This can be done, as proposed by Debinski’s group more than 10 years ago [32] by using a cocktail of combined therapies that can effectively bring the tumor ecosystem down. One such approach is by targeting multiple receptors in GBM, which will be described in more detail below.

## 4. Eph/Ephrin Receptor System in Gliomas 

Isolated from a hepatocellular carcinoma cell line, erythropoietin-producing-human (Eph)s are the largest family of receptor tyrosine kinases that bind to ephrins (eph receptor interacting ligands) [160]. They are important in cell adhesion, migration, guidance, and embryogenesis. Given their critical role in cell migration, they are also expressed in tumors and are active in wound healing and injuries [161,162,163,164,165]. Eph receptors bind to ephrin As and Bs and are internalized after ligand/receptor interaction and clustering in a matter of minutes. Since the ligands ephrin A and B are attached to the cell membrane, matrix metalloproteins are necessary to detach ligands from the cell surface and thereby establish a ligand/receptor interaction in addition to cell-cell interaction [166]. Overexpression of Eph receptors is quite ubiquitous among malignancies including gliomas, pancreatic, breast, ovarian, and other cancers [167].

The EphA2 receptor is over-expressed in ~60% of GBM tumors, and it is associated with grade increases in higher-grade gliomas, and with poor prognosis and survival [168,169,170]. Ephrin-B2 is expressed in astrocytomas; this expression along with expression of EphB4 increases with glioma grade [171]. The EphA7 receptor is present in tumor cells and endothelial cells of the vasculature surrounding the tumor [172]. Also, EphB3, B2, and B4 expression is increased in GBM compared to normal brain [173]. This makes Eph receptors attractive for novel targeted drug/therapy avenues.

Another Eph receptor, EphA3, is overexpressed in almost 60% of GBM patient samples [174] with increased presence in tumor initiating and infiltrating cells both in glioma [174] and leukemia [175], invasive rings, and scattered areas within the tumor and niches close to the blood vessels [174]. EphA3 is overexpressed in mesenchymal GBM, with EphA3 knockdown tumors exhibiting decreased tumorigenic potential [176]. Ephrin-A5 binds to the EphA2, EphB2, and EphA3 receptors [174]. Ephrin-A1 conjugated to a modified PE has been devised in the Debinski lab to target EphA2-overexpressing cells. The conjugate showed significant killing of GBM cells both in vitro (IC_50_ of 10^−11^ mol/L) and in vivo [177]. A conjugate of ephrinA5 and modified PE also showed high potency and specificity of tumor cells killing [174].

A study involving a cocktail of ephrin-A1 and IL-13 based cytotoxins has been extended to Phase I trials for glioma in dogs, and is showing extremely encouraging clinical effects [178]. Beta-emitting radionucleotide lutetium (^177^Lu) conjugated to monoclonal antibodies (mAb IIIA4) that target EphA3 receptors in mice implanted with U-251 MG GBM cells have been shown to prolong animal survival [176]. Similarly, CAR T-cell therapy that selectively targets EphA2 in an orthotropic xenograft SCID mouse model has shown promising results [179]. Combined expression of EphA2, EphA3, EphB2, and IL-13RA2 is observed in almost 100% of patients with glioblastoma patients. Furthermore, expression of these receptors is also present in tumor-infiltrating cells, tumor-initiating cells or GSCs, and neovasculature [180]. Therefore, a cytotoxic agent that simultaneously targets these four receptors could be powerful in destroying the tumor ecosystem of GBM without allowing it to recover and cause recurrence. Ferluga et al. has recently demonstrated such a multi-targeted approach in treating GBM by targeting multiple Eph receptors at once [174]. 

Desatinib is a multi-targeted kinase inhibitor that inhibits EphA2 and EphB2 receptors and has been tested against breast and ovarian cancer cells [181,182]. Doxazosin is a small molecule inhibitor of EphA2 and EphA4 that suppresses cell migration in glioma, breast cancer, and prostate cancer cells after internalization of receptors [183,184]. While it remains unclear how doxazosin inhibits migration, it is hypothesized that it activates receptor tyrosine kinases involved in inhibition of migration [185]. Eph receptors affect the migration signaling pathway by balancing the Rho versus Rac and Cdc42 pathways [186]. Enhancing the Rho pathway increases acto-myosin contractility within the cell, which explains the ligand-dependent stimulation of cell rounding upon Eph activation [184,187,188]. Suppression of cell migration of cells following doxazosin-Eph interaction may also stem from potential activation of Rho pathways that enhance cell rounding and suppress cell migration. Inhibitors like desatinib and doxazosin could be used in conjunction with other agents to increase anti-tumor activity.

## 5. EGFR/EGF Receptor System in Gliomas 

One of the most studied receptor systems in brain tumors is the EGFR/EGF receptor system [115,189,190,191]. The human EGFR receptor (HER2) family of receptor includes EGFR, HER2/neu, HER3 and HER4. They all contain similar structures except for the absence of tyrosine kinase in HER3. All bind to HER2 with extracellular ligands except for HER2. EGFR is a 170-kD transmembrane protein receptor with an extracellular domain, a transmembrane domain, and a tyrosine kinase domain. The N-terminus containing the extracellular domain creates a cleft of two regions that allow ligands to bind to the receptor [192]. *EGFR* is amplified in 40–60% of patients with GBM [191,193], while the protein is overexpressed in 38% of patients. However, EGFR expression is not specific to glioma cells [193]. About 30–50% of *EGFR* amplifications contain *EGFRvIII* mutations where the coding region of the extracellular domain is absent (deletion of exons 2–7). The mutated protein is expressed in fewer than 20% of patients with GBM, and is very heterogenous and patchy. The absence of the extracellular domain of the EGFR in EGFRvIII causes constitutively active tyrosine kinase activity, making them more invasive and antiapoptotic [190]. EGFRvIII-containing gliomas have enhanced Ras activity, Akt/PI3k signaling, and increased expression of VEGF and IL-8 [194]. Cells containing EGFRvIII also tend to express B-cell lymphoma extra-large (Bcl-XL) protein, which enhances their apoptotic ability [195].

Multiple groups have used EGFR and EGFRvIII receptors in glioma as targets for therapy [189]. Unfortunately, no clinical trials involving EGFR and EGFRvIII have shown significant clinical effects in patients. siRNA have been targeted towards EGFR that inhibited EGFR-expressing cells in vitro [196]. Treatment arrested glioma cells in the G2/M phase, significantly inhibiting cell growth in vitro. The siRNA also reduced tumor growth in vivo [197]. Erlotinib, an EGFR inhibitor, was not able to show significant effects in multicenter Phase I/II trial [198]. However, there was a strong correlation of PTEN and EGFRvIII co-expression with the outcome of erlotinib treatment. Not surprisingly, tumors that were PTEN-negative did not respond to the EGFR inhibitor [199,200]. 

Another inhibitor of EGFR, geftinib, was evaluated in Phase I/II clinical trials and showed no significant response in patients with newly diagnosed with GBM [201]. The monoclonal antibody cetuximab, which targets the extracellular domain of the EGFR was investigated in Phase I/II studies [202]. While only a few patients responded to the therapy, combining bevacizumab and irinotecan showed improved responses in patients [203]. The study showed 6-month progression- free survival in 46% of patients; 57% of patients showed a partial response, and 6-month overall survival was 77%. However, the study was terminated because one patient developed severe hemorrhage in the central nervous system, and another developed intestinal perforation and died. Nimotuzumab was evaluated in Phase I/II clinical trials for malignant glioma, and was well tolerated in patients [204]. Similarly, TGF-α conjugated to PE38, also called TP-38, has been evaluated in Phase I/II clinical trials. One patient demonstrated a complete response with 83-week survival, and 3 of 15 patients who received therapy showed radiographic response [205]. 

EGFRvIII-targeted CAR T-cell therapy is also being evaluated by Rosenberg’s lab at the National Cancer Institute (NCT01454596) and at Novartis and the University of Pennsylvania (NCT02209376) [206,207]. In the phase I study, EGFRvIII CAR T-cells were well tolerated by patients and did not show signs of off-target toxicity. A single intravenous dose of CAR T-cells was used and cytokine release syndrome was not observed. The CAR T-cells homed to tumor cells as shown by immunohistochemistry [206]. HER2-specific CAR T-cells have shown antitumor activity with increased production of stimulatory cytokines like IL-2 and interferon-γ. This antitumor response resulted in tumor regression in mice containing xenograft GBM cells. Similarly, HER2 CAR Cytomegalovirus-specific T therapy was evaluated in a phase I clinical trial with 16 patients. One patient showed partial response with about a 62% decrease in tumor volume over 8 months. The median survival of patients was 11.6 months after infusion of the therapeutic agent (NCT02442297 and NCT0110905) [208,209].

## 6. Other Receptors/Targeted Therapies

Other receptors used to treat gliomas include vascular endothelial growth factor receptor (VEGFR) [210], platelet-derived growth factor receptor (PDGFR) [211], transforming growth factor receptor (TGFR) [212], fibroblast growth factor receptor (FGFR) [213], c-Met receptor [214], IL-4 receptor [215], urokinase-type plasminogen receptor (uPAR) [216], g-protein coupled receptors (GPCR) [217], transferrin [218], lactoferrin [219], low density lipoprotein receptor (LDL-R) and low density lipoprotein receptor-related protein (LRP1) [220], tenascin, folate receptor (FOLR) [221], and integrin-mediated targeting systems [222]. The VEGFR targeted antibody bevacizumab (Avastin) was FDA-approved to treat recurrent GBM [223], based on durable radiologic responses in a Phase II trial [224]. Patients showed a significantly longer progression-free survival time (median survival of 46.8 months) when treated with bevacizumab, compared to the expected survival of 16.3 months with standard temozolamide and radiation treatments [225]. However, it failed to show efficacy in GBM patients in all phase III trials [223].

A Phase III trial investigated the efficacy of cilengitide, an RGD peptide that targets the integrin combined with standard therapy in patients with newly diagnosed GBM. Results from Phase I/IIA studies had shown that patients with MGMT promoter methylation status responded better to cilengitide [226]. In the Phase III trial, 3471 patients were screened for MGMT status; 926 patients had a MGMT promoter and received the treatment. Overall, cilengitide with temozolomide and radiation therapy did not improve clinical outcomes [227]. The lack of clinical response with therapies targeted at individual receptors reaffirms the notion that glioma treatment should simultaneously address various facets of the tumor to confer significant, long-lasting clinical impact. 

Tyrosine kinase inhibitors against VEGFR, including cediranib, sorafenib, sunitinib, and vandetanib, have been tested in Phase I/II trials and their efficacy is now being tested for GBM [228]. In a phase III clinical trial (NCT00777153) with 325 patients, the efficacy of cediranib was tested alone and in combination with lamoustine, but with no significant improvement in progression-free survival occurred in either treatment arm [229]. Sorafenib plus emozoalide was evaluated in 43 patients with GBM in a phase II trial and was safe. Six-month progression-free survival was observed in 26% of patients with a median survival of 7.4 months [230]. A phase II evaluation of sunitinib in children with high-grade glioma and ependymoma (NCT014626695) showed that the treatment was well tolerated, but did not result in significant antitumor activity [231]. Tyrosine kinase inhibitors for PDGFR include imatinib, which showed limited activity against glioma in Phase I and II clinical trials [232]. Desatinib, another inhibitor of PDGFR, showed no clinical benefits for recurrent GBM in a Phase II trial (NCT00423735) [233]. Brivanib and XL184, two other tyrosine kinase inhibitors for FGFR and c-Met receptors, respectively, were tested in preclinical and Phase I/II trials, respectively [234]. 

Targeting the transferrin receptor with Tf-CRM107 immunotoxin was evaluated in Phase I/II/III trials using CED. This was the first clinical trial to test the efficacy of an immunotoxin in GBM. Diphtheria toxin with a point mutation (CRM107) was conjugated with transferrin by a thioester bond to form a cytotoxin-protein conjugate that targets the transferrin receptor in glioma. In vitro analysis toxin showed that the unconjugated toxin was 1000–100,000 times less toxic to glioblastoma cell lines; its IC_50_ value ranged from 2.6 × 10^−12^ to 6.5 × 10^−11^ mmol/L. In a Phase I trial, 18 patients were treated, 9 out of 15 patients showed >50% decrease in tumor and 2 patients showed complete responses [235]. While 7 of 44 patients with recurrent GBM showed a partial response to Tf-CRM107 immunotoxin, the Phase III trial was stopped due to the lack of significant efficacy [235]. The transferrin receptor is expressed in normal endothelium, which may limit the therapeutic window of the immunotoxin.

## 7. Immune Checkpoint Inhibitors

A potential new facet of glioma therapy includes immune checkpoint inhibitors. GBM cells consistently work to maintain and generate an immunosuppressive environment. Expression of receptors that bind to cytotoxic T-cells and inhibit their action is common in GBM. Specifically, expression of galectin-9 that binds to T-cell immunoglobulin and mucin domain 3 (TIM-3) [236]; Fas ligand (FasL) that induces apoptosis of lymphocytes [237], B7 which is immunosuppressive to CTLA-4 [238], and programmed death ligand 1 (PD-L1) which is immunosuppressive to programmed death 1 (PD-1) expressing T-cells are significantly high in glioma cells [239]. The expression of PD-L1 in particular is higher in glioma-infiltrating cells than in glioma cells. GBM also overexpress major histocompatibility complex class I (MHCI) molecule human leukocyte antigen G (HLA-G) which inhibit natural killer cell and cytotoxic T-cell responses. Synthesis of anti-inflammatory cytokines including chemokine (C-C motif) ligand 2 (CCL2), tumor growth factor β (TGFβ), interleukin 10 (IL-10) that recruit regulatory T cells and myeloid derived suppressor cells (MDSC), and tumor-associated macrophages (TAMs) that inhibit T cell responses and promote tumor progression are also very common in GBM [240]. Downregulation of human leukocyte antigen I (HLAI) molecules, which allow tumor cells to evade cytotoxic T cells, has been shown in GBM compared to LGGs [241]. Immunomodulation of the glioma environment may therefore provide significant therapeutic benefits. 

T cells express CTLA-4 that would be cytotoxic to tumor cells. However, tumor cells express B7 that bind to CTLA-4 and inhibit these cytotoxic effects. Ipilimumab is a mAb against CTLA-4 that was approved by FDA for melanoma in 2011. It has shown benefits to patients with brain metastasis as a single therapy and combined with radiation therapy [242]. CTLA-4 blocking antibodies improved survival in mice. Similarly, anti-CTLA-4 antibodies in conjunction with IL-12, which promotes survival of T cells, was effective against GL261 glioma cells [243]. Furthermore, the CTLA-4 antibody plus cytokine granulocyte macrophage colony stimulating factor was more effective than the treatments alone [244].

PD-1 is expressed in T cells, TAMs, vascular endothelial cells, and neurons of the tumor microenvironment. It binds to PD-L1 and programmed death ligand 2 (PD-L2), which are overexpressed in GBM cells. Inhibiting PD-L1/PD-1 interaction allows cytotoxic T cells to effectively kill tumor cells [245]. Pembrolizumab and ivolumab are PD-1 inhibitors approved by the FDA for the treatment of melanoma in 2014, with subsequent approvals for treatment in seven more cancers. A Phase III trial of nivolumab and ipilimumab was initiated for GBM in 2014, but failed completely [246]. A Phase I trial of ipilimumab plus nivolumab (a PD-1 inhibitor) is ongoing for patients after resection of recurrent glioblastoma (NCT03233152) [247]. Other PD-1/PD-L1 interaction inhibitors include AMP-224, MPDL32804, MSB0010418C, and MEDI4736 for other hematologic and solid tumors [248]. Anti-CTLA-4, anti-PD-1 and 1-methylthryptophan (1-MT) significantly enhanced survival and lowered the number of regulatory T cells compared to monotherapies [249]. 

TIM-3 is a glycoprotein expressed in T-cells containing an extracellular IgG and mucin domain that binds to Galectin-9. TIM-3 binds to galectin-9 on tumor cells and suppresses immune response to GBM cells. TIM-3 and LAG-3 are also highly expressed other malignancies, e.g., non-small-cell lung carcinoma and melanoma [250]. Expression of TIM-3 is also elevated in CD4^+^ and CD8^+^ T cells of glioma patients compared to healthy counterparts [251]. In in vivo studies, anti-PD-1, anti-TIM-3 and radiation synergistically regressed glioma in mice [252]. 

Lymphocyte activation gene 3 (*LAG-3*) binds to MHC class II antigens and works together with PD-L1 to suppress the activation and expansion of T cells. LAG-3 is highly expressed in tumor- infiltrating lymphocytes in patients with fibrosarcoma, colorectal cancer, and melanoma [253]. A Phase I trial in patients with recurrent GBM (NCT02658981) testing combined therapy of an anti-LAG-3 monoclonal antibody and nivolumab is ongoing [55]. 

Immunostimulatory cytokines have also been used to treat glioma. IL-2 was first tested in 1986 [254]. A Phase I trial of suicide gene therapy of herpes simplex virus type 1-thymidine kinase and retrovirus-producing IL-2 shown partial response with minimal side effects [255]. Subcutaneous IL-4 injections plus herpes simplex virus type 1-thymidine kinase increased interferon γ production in a rat model, and is being tested in a Phase I clinical trial [256]. Similarly, administration of interferon γ enhanced efficacy of temozolomide [257]. Liposomal delivery of interferon γ reduced tumors by half in a Phase I clinical trial, and Ad-hIFNγ has shown safety and tumor cell apoptosis [258].

Given that checkpoint inhibitors target immune responses and are critical in maintenance of the tumor ecosystem, checkpoint inhibitors and immunostimulatory cytokines are appealing as treatments for GBM in conjunction with chemotherapy, radiation therapy and a myriad of receptor-targeted therapies like cytotoxins and viral gene therapy. However, substantial clinical effects to back this notion have not yet been observed. Furthermore, although rare, immune checkpoint inhibitors have been associated with immune-related adverse events. For example, in a single-center review of over 500 patients treated with the immune checkpoint inhibitors ipilimumab and nivolumab in 2005–2017, 5 patients had immune-related adverse events (0.95% incidence rate) [259]. These ranged from sensory neuropathy and transverse myelopathy, to oculomotor nerve palsy and posterior reversible encephalopathy syndrome, with more severe events in patients receiving combination immunotherapy.

## 8. Viral/Genetic Therapies 

Development of vectors that can successfully deliver genes into the tumor has allowed testing of genetic therapy for glioma. Delivery of gene therapy is broadly based on viral vectors (adenovirus, herpes simplex, measles, reovirus, and poliovirus) [260] and non-viral vectors like liposomes and nanoparticles. Virus-based therapies can have multiple therapeutic effects including tumor cell death, increased anti-tumor immune response, and release of damage- associated molecules like high mobility group box 1 (HMGB1) [261,262]. Use of suicide gene therapy, where an inactive prodrug ganciclovir thymidine kinase is converted into a toxic metabolite to cause tumor apoptosis, has been investigated in GBM [263]. While the results from Phase I/II preclinical trials were promising, a large Phase III trial of herpes simplex virus type 1-thymidine kinase/gancyclovir plus surgery and radiation therapy did not significantly improve survival among patients newly diagnosed with GBM [264]. A combined cytotoxic and immune-stimulatory therapy for glioma is underway. Phase I trial (NCT01811992) of adenovirus mediated delivery of FMS like tyrosine kinase 3 ligand (Flt3L, which stimulates the migration of dendritic cells) along with Ad tyrosine kinase and ganciclovir increased survival of mice, and generated tumor-specific T-cell responses and long-term cell memory, along with release of HMGB1 by dying tumor cells [265].

Over 20 oncolytic virus candidates have been tested in clinical trials to treat GBM. Several oncolytic viruses have demonstrated safety in Phase I clinical trials for glioma, including reovirus [266], oncolytic measles [267], conditionally replicating adenoviruses (CAds) [268] and oncolytic herpes simplex virus [269]. ONYX-015 (adenovirus mutant dl1520) has been shown to be safe when injected intracranially to treat glioma. This virus has been engineered to divide in cells lacking p53 [270]. A Phase I/II clinical trial of Ad 5Delta 24-RGD and DNX2401 is underway in patients with recurrent GBM (NCT01582516) [108]. Similarly, DNX2401 (NCT02197169) expressing interferon γ was tested in a Phase I trial for patients with recurrent GBM [271]. Other viruses tested for GBM include the M protein mutant (M51R) and vesicular stomatitis virus, which showed selectivity in killing U87 GBM cells versus normal brain cells [272]. 

Some viral constructs take advantage of the overexpression of CD46, which can serve as a measles virus receptor; the laminin receptor, which could serve as a sindbis virus receptor; and CD155, which is overexpressed in glioma cells and can serve as a receptor for poliovirus. Furthermore, viruses have been designed to specifically target receptors that are overexpressed in glioma cells including PDGFR, IL-13RA2, and EGFRvIII [273]. Presently, a Phase I clinical trial using carcinoembryonic antigen-expressing measles virus is underway for GBM. The study is designed to administer a starting does of 10^5^ TCID_50_ to be escalated to 2 × 10^7^ TCID_50_. Until now, no dose-limiting toxicity has been established [110,267,273,274,275]. These viruses have enhanced efficiency of therapeutic gene transduction compared to non-replicating viruses. However, consistent clinical benefits of these viruses are yet to be established.

## 9. Conclusions

Treating brain tumors like gliomas is extraordinarily difficult. Their location and infiltrative nature, the presence of the BBB, molecular, genetic and phenotypic heterogeneity, the poorly developed but well vascularized immunosuppressive environment, and resistance to therapy are some of the major challenges facing the treatment of brain tumors. Moreover, brain tumors function as an ecosystem with highly interconnected networks that are able to compensate for each other and bounce back after treatment. Hence, the clinical prognosis for brain tumors has not changed substantively over the last few decades. However, there have been notable advancements in antigen-specific targeting, viral and genetic silencing, immune checkpoint inhibitors, peptide and dendritic cell vaccines, CAR T-cell therapy, chemotherapy and radiation therapy, in conjugation with effective drug delivery systems like CED (Table 1). Combinatorial therapies damage multiple facets of the tumor ecosystem, giving hope to eventual cure of brain tumors. Similarly, neoantigen screening of patients using next-generation sequencing would allow treatments to be individually tailored [276]. Furthermore, the antigen profile of tumors could be altered after exposure to ionizing radiation/surgery. Hence, detailed studies of changes in antigen expression before and after radiation or chemotherapy would help predict therapeutic outcomes and modify treatment plans accordingly for maximum efficacy. Also, deciphering the mechanisms of immunoediting could lead to efficient immunotherapies with superior clinical benefits. In addition, effective and innovative monitoring mechanisms that can critically evaluate the efficacy of single and combinatorial therapies, along with CT and MRI imaging, could help enhance the success of therapeutic strategies for brain tumors. Similarly, technologies like the IsoChip and IsoLight [277,278] that can eventually predict the outcome of potential therapies ex vivo before being introduced into patients could provide powerful tools to better understand and treat this devastating disease.

## Figures and Tables

**Table 1:** An overview of the targeted therapies used to treat glioblastoma.

Type of Therapy	Therapy	TSA/TAA Target (s)	Reference (s)
Peptide vaccines	Poly ICLC	IL-13RA2, Wilms Tumor 1, Survivin	[50]
	CDX-110	EGFRvIII	[116]
	Poly ICLC	IL-13RA2, EphA2, Survivin	[119]
	Cilengitide	Integrin	[226]
Liposomes/Nanoparticles/Nanosheets	IL-13 based Liposome	IL-13RA2	[104,105]
	IL-13 based Nanoparticles	IL-13RA2	[106,107]
	Transferrin based Nanoparticles	Transferrin receptor	[218]
	Lactoferrin based Nanoparticles	Lactoferrin receptor	[219]
Radiotherapy	EphA3 mAb(IIIA4) Radiolabelled with (177 Lu)	EphA3	[176]
	[64Cu]Pep-1L	IL-13RA2	[63,74]
Antigen pulsed dendritic cells	Multiple Epitope Pulsed Dendritic Cells (ICT-107)	IL-13RA2, MAGE-1, TRP-2, gp-100, EGFR, AIM-2	[114]
Antibodies	Cetuximab	EGFRvIII	[202]
	Bevacizumab and Irinotecan	VEGF	[203]
	Nimotuzumab	EGFR	[204]
	Bevacizumab	VEGF	[223]
Immunotoxins	Transferrin-CRM 107	Transferrin	[235]
	ephrin A1 based Cytotoxin	EphA2	[177]
	TGF-α based Cytotoxin	EGFR	[205]
	IL-4 based Cytotoxin	IL-4RA	[215]
	Amino-terminal Fragment of uPA based Cytotoxin	uPAR	[216]
	IL-13 and ephrin A1 based Cytotoxins	IL-13RA2, EphA2	[178]
	ephrin A5 based Cytotoxins	EphA2, EphA3, EphB2	[174]
	IL-13 based Cytotoxin	IL-13RA2	[65]
CAR T-cell therapy	CAR T-cells targeting EGFRvIII	EGFRvIII	[206]
	CAR T-cells targeting HER2	HER2	[208,209]
	CAR T-cells targeting EphA2	EphA2	[179]
	IL-13.E13K.R109K CAR T cells	IL-13RA2	[138]
	TanCARs	HER2, IL-13RA2	[150]
	GRm13Z40-2	IL-13RA2	[56,129,137]
	IL-13(E13Y)-zetakine CAR T cells	IL-13RA2	[125]
	IL-13BBζ-CAR T cells	IL-13RA2	[130]
	IL-13 (E13K, E13Y, E13K.K105R, E13Y.K105R) based CAR T-cells	IL-13RA2	[131]
	IL-13RA2 with Herpes Simplex Virus and PET reporter gene	IL-13RA2	[134,135]
Viral/Genetic Therapy	Delta-24-RGD (DNX-2401) Oncolytic Adenovirus	αvβ3 and αvβ5 Integrins	[108]
	Ad.mhIL-4.TRE.mnhIL-13-PE) Adenovirus	IL-13RA2	[113]
	R5141, Recombinant Herpes Simplex Virus-1	IL-13RA3	[111]
	DARPin-targeted Measles Virus	HER2	[112]
	LU-13, Adenovirus	IL-13RA2	[109]
	IL-13 based Lentiviral Vectors	IL-13RA2	[110]
	Oncolytic Measles Virus	CD46	[267,274,275]
	Reovirus	N/A	[266]
	Conditional Replicating Adenovirus (CAd)	Coxsackie and adenovirus receptor	[268]
	Oncolytic Herpes Simplex Virus	CD133 and CD111	[269]
	ONYX-015, Recombinant Adenovirus	Coxsackie and adenovirus receptor	[270]
	Oncolytic Vesicular Stomatitis Virus	Death receptor	[272]
	IL-4 and Herpes Simplex Virus Thymidine Kinase	CD133 and CD111	[256]
	Herpes Simplex Virus-Thymidine Kinase Ganciclovir (HSV-TK/GCV)	CD133 and CD111	[263]
Immune checkpoint inhibitors	Ipilimumab	CTLA-4 on T-cells	[242]
	Nivolumab and Ipilumumab	PD-1 and CTLA-4 on T-cells respectively	[247]
	Anti TIM-3	TIM-3 on T-cells	[252]
	Anti LAG-3 and Nivolumab	LAG-3 and PD-1 on T-cells respectively	[55]
	anti-CTLA-4 mAb (9D9, IgG2b)	CTLA-4 on T-cells	[243]
	ani-CTLA-4mAb and Granulocyte-Macrophage Colony Stimulating Factor (GMCSF)	CTLA-4 on T-cells	[244]
Tyrosine kinase inhibitors	Cediranib	VEGFR	[229]
	Sorafenib	VEGFR	[230]
	Sunitinib	VEGFR	[231]
	Vandetanib	VEGFR	[228]
	Imatinib	PDGFR	[232]
	Desatinib	PDGFR	[233]
	Brivanib	FGFR	[234]
siRNA based Therapy	Lipid Nanocapsules	EGFR	[196]
	Transfection of siRNA	EGFR and β-catenin	[197]
Small molecule inhibitors	Erlotinib	EGFR	[198]
	Geftinib	EGFR	[201]

## Abbreviations

1-MT	1-Methylthryptophan
ACT	Adoptive Cell Transfer
ACTL8	Actin Like Protein 8
AIM-2	Absent In Melanoma 2
ALL	Acute Lymphoblastic Leukemia
BAGE	B Melanoma Antigen
BBB	Blood-Brain Barrier
Bcl-XL	B-cell Lymphoma Extra-Large
BORIS	Brother Of The Regulator Of Imprinted Sites
CAds	Conditionally Replicating Adenoviruses
CAR T	Chimeric Antigen Receptor T-cells
CB	Cintredekin Besudotox
CCL2	Cytokines Including Chemokine (C-C motif) Ligand 2
CD	Cluster of Differentiation
CED	Convection-Enhanced Delivery
CNS	Central Nervous System
CT	Computerized Axial Tomography
CT 45	Cancer/Testis Antigen 45
CTAGE1	Cutaneous T-cell Lymphoma Associated Antigen 1
CTL-4	Cytotoxic T-lymphocyte-Associated Protein 4
D2	Domain 2
DARPins	Designed Ankyrin Repeat Proteins
DDX43	Dead-Box Helicase 43
E	Glutamic Acid
EGF	Endothelial Growth Factor
EGFR	Epidermal Growth Factor Receptor
Eph	Erythropoietin-Producing-Human
ephrin	Erythropoietin-Producing-Human Receptor Interacting Ligands
FasL	Fas ligand
FDA	Food and Drug Administration
FGFR	Fibroblast Growth Factor Receptor
FOLR	Folate Receptor
GAGE	G-Antigen
GBM	Glioblastoma
GFAP	Glial Fibrillary Acidic Protein
gp-100	glycoprotein 100
GPCR	G-Protein Coupled Receptors
HAGE	Helicase Antigen
HER-2	Epidermal Growth Factor Receptor
HLA-G	Human Leukocyte Antigen G
HLAI	Human Leukocyte Antigen I
HMGB1	High Mobility Group Box 1
IDH1	Isocitrate Dehydrogenase Gene 1
IFN γ	Interferon γ
IL	Interleukin
IL-13RA1	Interleukin-13 Receptor A1
IL-13RA2	Interleukin 13 Receptor Alpha 2
IL-4RA	Interleukin-4 Receptor A
K	Lysine
KNL1	Kinetochore Scaffold 1
LAG-3	Lymphocyte Activation Gene 3
LDL-R	Low Density Lipoprotein Receptor
LGG	Lower Grade Glioma
LRP1	Low Density Lipoprotein Receptor-Related Protein
MAGE	Melanoma Associated Antigen
MAPK	Mitogen-Activated Protein Kinase
MART	Melanoma-Associated Antigen Recognized by T cells
MDSC	Myeloid Derived Suppressor Cells
MGMT	Protein O6 Methylguanine-DNA Methyltransferase
MHC	Major Histocompatibility Complex
MHCI	Major Histocompatibility Complex Class I
MRI	Magnetic resonance Imaging
NLS	Nucleus Localizing Signal
OIP5	Opa-Interacting Protein 5
PD-1	Programmed Death 1
PDGFR	Platelet-Derived Growth Factor Receptor
PD-L1	Programmed Death Ligand 1
PD-L2	Programmed Death Ligand 2
PTEN	Phosphatase and Tensin Homolog
PTEN	Phosphatase And Tensin Homolog
RANO	Response Assessment in Neuro-Oncology
scFv	Small Chain Variable Fragment
SCID	Severe Combined Immunodeficiency
SP 17	Sperm Protein 17
SSX	Synovial Sarcoma X
STAT6	Signal Transducer And Activator Of Transcription 6
TAA	Tumor-Associated Antigens
TAM	Tumor-Associated Macrophages
TERT	Telomerase Reverse Transcriptase
TGFR	Transforming Growth Factor Receptor
TGFβ	Tumor Growth Factor β
T_H_2	T Helper 2
TIM-3	T-cell Immunoglobulin And Mucin Domain 3
TNFα	Tumor Necrosis Factor α
TP53	Tumor Protein 53
TRP-2	Tyrosine-Related Protein 2
TSA	Tumor-Specific Antigens
TT Fields	Tumor-Treating Fields
Upar	Low Density Lipoprotein Receptor
VEGFR	Vascular Endothelial Growth Factor Receptor
XAGE3	X-Antigen Family Member 3
Y	Tyrosine
